# Acromial morphology: reliability of computer tomography–based assessment and association with age: a study of 420 patients

**DOI:** 10.1016/j.jseint.2026.101745

**Published:** 2026-05-23

**Authors:** Yohan Gagnard, Florent Baldairon, Thomas Mereb, Geoffrey Ginot, Philippe Clavert

**Affiliations:** a CHU de Strasbourg, Hôpital de Hautepierre, Strasbourg, France; b Clinique du Sport Bordeaux-Mérignac, Mérignac, France; c Institut de Chirurgie Orthopédique et Sportive Alsace Centrale, Hôpital Albert Schweitzer, Colmar, France

**Keywords:** Acromion morphology, Basic science study, Validation of classification system using imaging, Subacromial impingement, CT, Radiography, Shoulder joint, Age factor

## Abstract

**Background:**

Acromial morphology is considered an important factor in the pathophysiology and development of subacromial impingement and rotator cuff disorders. The Bigliani classification remains widely used to describe acromial shape in both clinical practice and research, mainly because of its simplicity; however, its reliability on computed tomography (CT) imaging, including CT arthrography, has been poorly investigated. In addition, the relationship between acromial morphology and age remains unclear. The primary objective of this study was to assess the intrarater and inter-rater reliability of the Bigliani classification on CT imaging. The secondary objective was to analyze the association between acromial morphology and age.

**Methods:**

This was a single-center retrospective observational study. Five shoulder surgeons with different levels of experience independently assessed the morphology of 420 acromions using standardized slices derived from three-dimensional CT reconstructions. Acromial morphology was classified according to the Bigliani classification. All evaluations were performed in a blinded fashion.

**Results:**

Among the 2,100 evaluations performed, 28.7% of acromions were classified as type I, 59.7% as type II, and 11.6% as type III. The Bigliani classification demonstrated good intrarater reliability (κ = 0.61) but poor inter-rater reliability (κ_f_ = 0.32). A significant association was observed between age and acromial morphology.

**Conclusion:**

Even with optimized CT imaging, the Bigliani classification demonstrated good intrarater reliability but poor inter-rater reliability for the assessment of acromial morphology. A significant association between age and acromial morphology was identified. These results should be interpreted with caution, as this study was designed as a methodological evaluation of a widely used classification system rather than to establish clinical or causal relationships, with potential implications for clinical decision-making and research reproducibility.

Subacromial impingement syndrome, initially described by Neer,^28^ is one of the most common shoulder disorders.^26^ It is classically attributed to compression of the rotator cuff tendons within the subacromial space.^36^

However, the pathophysiology of subacromial impingement and the role of acromial morphology remain debated, and the clinical relevance of these concepts has been increasingly questioned in recent literature.

Acromial morphology is frequently considered a potential contributor to rotator cuff disorders,^4^^,^^8^^,^^26^^,^^34^^,^^36^ although a direct association remains debated in the literature.^6^^,^^5^^,^^7^^,^^9^^,^^11^^,^^13^^,^^15^^,^^16^^,^^21^^,^^23^^,^^31^^,^^32^^,^^41^^,^^42^

In 1986, Bigliani et al^2^ first proposed a three-type classification (Fig. 1) based on supraspinatus outlet radiographs to characterize acromial morphology: type I (flat), type II (curved), and type III (hooked). Despite concerns regarding its clinical utility and its reproducibility, this classification remains widely used in clinical practice and research.^3^^,^^15^^,^^17^^,^^31^^,^^33^ To address this variability, alternative classifications have been proposed, but their clinical use remains limited.^9^^,^^27^^,^^30^^,^^33^^,^^38^^,^^39^

**Figure 1:**
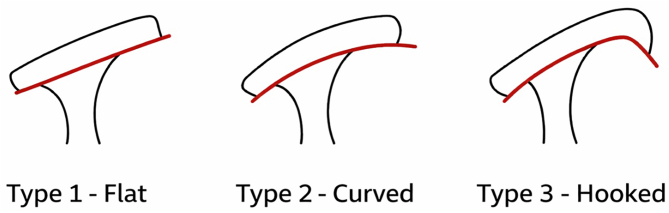
Bigliani classification^2^ of acromial morphology: type I (flat), type II (curved), and type III (hooked).

Therefore, evaluating its reliability, particularly using modern imaging techniques, remains relevant.

Data regarding the reproducibility of the Bigliani classification on three-dimensional (3D) computed tomography (CT and CT arthrography [CTA]) reconstructions are limited, although CT imaging is frequently used for the evaluation of shoulder pathology.^14^

Age is a well-recognized factor in the development of rotator cuff tears.^4^^,^^36^ However, the relationship between age and acromial morphology remains controversial, with conflicting results reported in radiographic, magnetic resonance imaging, cadaveric, and histological studies.^12^^,^^22^^,^^29^^,^^35^^,^^37^^,^^40^

In this context, evaluating acromial morphology should be approached as a methodological issue related to the validity and reproducibility of commonly used classification systems, rather than solely as a determinant of clinical decision-making.

The primary objective of this study was to assess the intrarater and inter-rater reliability of the Bigliani classification on CT imaging. The secondary objective was to analyze the relationship between acromial morphology and age.

This study does not aim to support the clinical use of the Bigliani classification, but rather to critically evaluate its reliability when applied to standardized 3D CT reconstructions.

We hypothesized that CT-based assessment of acromial morphology according to the Bigliani classification would demonstrate satisfactory reliability and that acromial morphology would vary with age.

## Materials and methods

### Sampling method

This was a single-center retrospective observational study.

All shoulder CT and CTA examinations performed at the Strasbourg University Hospital between January 2, 2009, and December 11, 2023, were identified regardless of clinical indication, yielding a total of 9,185 examinations.

From this database, the most recent examinations were selected consecutively and stratified into predefined consecutive 5-year age groups based on patient age at the time of imaging.

To ensure adequate representation across age groups and sufficient statistical power for reliability analyses, a total of 420 patients were included a priori.

Inclusion criteria were patients aged 20 to 89 years who underwent shoulder CT or CTA at the Strasbourg University Hospital during the study period.

Exclusion criteria included insufficient image quality, inability to perform 3D CT reconstruction, multiple examinations for the same patient within the same age group, a history of acromial fracture, prior surgery incompatible with acromial analysis (shoulder arthroplasty, acromioplasty, proximal humeral osteosynthesis, scapulohumeral arthrodesis), or any condition preventing reliable assessment of acromial morphology (severe acromioclavicular osteoarthritis, humeral head osteonecrosis, glenohumeral dysplasia).

### Computed tomography imaging and image processing

CT and CTA examinations were analyzed using 3D reconstructions generated with Xplore Console PACS software via the “Reformat” function from volumetric data.

For each examination, the scapula was isolated from surrounding bony structures (clavicle, humerus, rib cage, and sternum).

In CTA examinations, intra-articular contrast material was removed to allow optimal visualization of bony contours. Each scapula was then oriented to obtain a strict glenoid profile, ensuring standardization of the evaluation plane.

**Figure 2:**
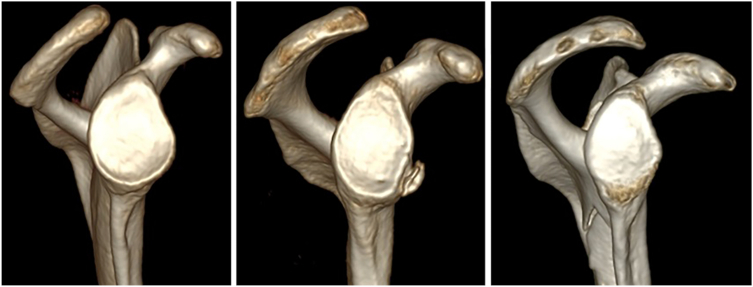
Three-dimensional reconstructions of acromions classified as type I, type II, and type III according to the Bigliani classification.

The resulting image was saved in .dcm and .jpg formats and anonymized for blinded analysis (Fig. 2).

### Raters

Five shoulder surgeons with different levels of experience participated in the analysis: 1 resident, 2 fellows (clinical assistants), 1 attending surgeon, and 1 senior academic surgeon.

No formal calibration session was performed prior to the evaluation. Raters were provided with the standard definitions of the Bigliani classification. Each rater independently evaluated all 420 images during a first reading session.

To assess intrarater reliability, all raters repeated the analysis six months after the initial reading, blinded to their previous assessments. This second reading was used exclusively for intrarater reliability analysis.

### Acromial morphology assessment

Acromial morphology was assessed using the Bigliani classification, which distinguishes 3 types: type I (flat), type II (curved), and type III (hooked).

Classification was performed visually using standardized images derived from the 3D CT reconstructions.

### Blinding procedure

All evaluations were performed in a blinded manner. Raters had no access to patients' clinical data or to the assessments of other raters. They were also blinded to their own previous results during repeated analyses.

### Statistical analysis

To address the primary objective, cross-tabulations were used to compare ratings between raters. Intrarater and inter-rater agreement was assessed using Cohen kappa coefficient (κ) for each pair of raters or for paired observations over time for the same rater.

Overall interrater agreement was assessed using Fleiss kappa coefficient (κ_f_), which evaluates agreement among more than 2 raters. Overall intrarater agreement was calculated by averaging individual kappa coefficients.

Interpretation of agreement coefficients was based on the Landis and Koch classification.^20^

To address the secondary objective, multinomial logistic regression models were used to analyze the Bigliani classification (categorical dependent variable) as a function of patient age (independent variable). This approach allowed estimation of the probability of belonging to each acromial type (types I, II, and III) according to age.

To account for interrater variability, analyses were performed separately for each rater, resulting in distinct models. For each model, odds ratios (ORs) and their 95% confidence intervals were calculated for a 1-year increase in age, comparing types II and III with type I.

A global test of the age effect was performed across all 3 categories of the classification, and exact *P* values associated with ORs and age inclusion in the models were reported.

Finally, a global analysis was conducted using a latent class model to integrate all evaluations and obtain a consensus estimate of Bigliani classification as a function of age.

## Results

### Study population

**Figure 3:**
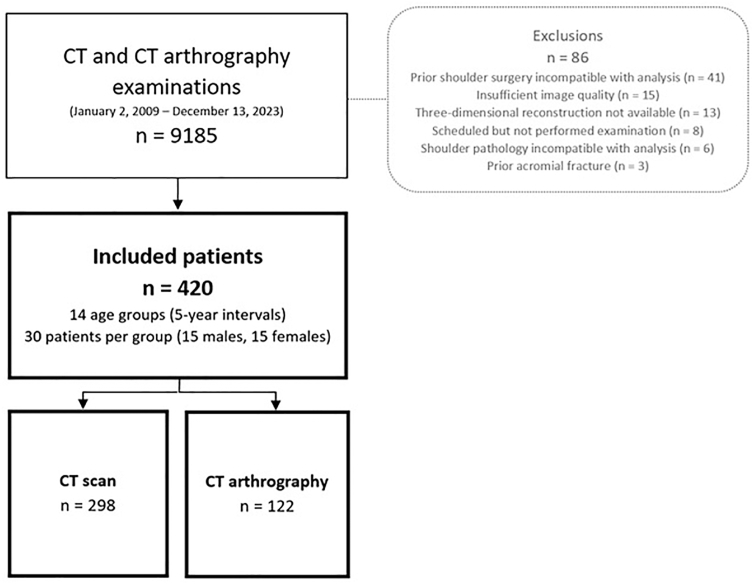
Flowchart of patient selection and inclusion criteria. *CT*, computed tomography.

Among the 9,185 examinations identified in the initial database, 420 patients were included and allocated into 14 age groups of 30 patients each, corresponding to 5-year age intervals at the time of imaging. A total of 86 examinations were excluded according to the predefined criteria (Fig. 3).

### Patient characteristics

The characteristics of the study population are summarized in Table I. The median age at the time of imaging was 55 years (interquartile rane, 38-72). CT was performed in 298 patients (71%), and CTA in 122 patients (29%).

**Table I:** Baseline characteristics of patients.

Categorical data are presented as numbers (percentage). Age is presented as mean (standard deviation) and ranged from 20 to 89 yr.	
Variable	Overall sample (*N = 420*)
Age, *yr*	54.4 ± 20.2
Sex	
Male	210 (50)
Female	210 (50)
Imaging modality	
CT scan	298 (71)
CT arthrography	122 (29)
Shoulder side	
Left	226 (54)
Right	194 (46)

*CT*, computed tomography.

### Indications for imaging

The 3 most frequent indications for CT imaging were, in descending order, fractures of the proximal humerus (36%), rotator cuff pathology (16%), and anterior shoulder instability (13%).

### Descriptive analysis of ratings

A total of 2,100 initial ratings were performed, corresponding to the evaluation of 420 images by 5 raters at the first reading.

Among these ratings, 28.7% of acromions were classified as type I, 59.7% as type II, and 11.6% as type III. Complete agreement across all 5 initial ratings was observed for 64 acromions (15.2%).

Overall, type II acromions were the most frequently assigned across all age groups.

The distribution of acromial types varied among raters, with proportions ranging from 14% to 45% for type I, from 49% to 81% for type II, and from 5% to 24% for type III.

### Intrarater reliability

Intrarater reliability was assessed for all raters by comparing evaluations performed six months apart on the same 420 patients.

Cohen kappa coefficients for individual raters ranged from 0.41 to 0.75, indicating moderate to good agreement (Table II). Overall intrarater reliability, calculated as the mean of individual kappa coefficients, was good (κ = 0.61).

**Table II:** Intrarater kappa (95% confidence interval).

Rater A	Rater B	Rater C	Rater D	Rater E	Mean
0.75 (0.69-0.81)	0.57 (0.49-0.64)	0.62 (0.54-0.69)	0.41 (0.33-0.50)	0.69 (0.61-0.75)	0.61

Global intrarater reliability is reported as the mean kappa value.

### Inter-rater reliability

Cohen kappa coefficients calculated for each pair of raters ranged from 0.21 to 0.43, corresponding to fair to moderate agreement (Table III). None of the confidence intervals included zero.

**Table III:** Interrater kappa (95% confidence interval).

Raters A/B			
0.43 (0.36-0.51)			

Raters A/C	Raters B/C		

0.22 (0.15-0.29)	0.21 (0.14-0.28)		

Raters A/D	Raters B/D	Raters C/D	

0.42 (0.35-0.49)	0.39 (0.31-0.47)	0.29 (0.20-0.37)	

Raters A/E	Raters B/E	Raters C/E	Raters D/E

0.31 (0.24-0.38)	0.37 (0.29-0.44)	0.22 (0.15-0.29)	0.38 (0.30-0.46)

Overall agreement among the 5 raters, assessed using Fleiss kappa, was low (κ_f_ = 0.32; *P* < .001).

Analysis by acromial type showed moderate agreement for type I acromions (κ_f_ = 0.41; *P* < .001) and low agreement for type II (κ_f_ = 0.26; *P* < .001) and type III acromions (κf = 0.26; *P* = .003).

### Association between acromial morphology and age

Multinomial logistic regression models demonstrated an association between age and Bigliani classification for several raters. ORs for a 1-year increase in age were calculated for each rater (Table IV). The global effect of age was statistically significant for 4 of the 5 raters.

**Table IV:** Logistic regression model for each rater: odds ratios per 1-year increase in age for acromions type 2 compared to type 1 (2/1) and type 3 compared to type 1 (3/1).

Rater	2/1		3/1	
	OR (95% CI)	*P* value	OR (95% CI)	*P* value
Rater A	1.02 (1.01-1.03)	<.001	1.04 (1.02-1.05)	<.001
Rater B	1.01 (1.00-1.02)	.026	1.00 (0.98-1.01)	.857
Rater C	1.01 (0.99-1.02)	.32	1.02 (1.00-1.05)	.076
Rater D	1.02 (1.01-1.03)	.003	1.00 (0.98-1.03)	.847
Rater E	1.02 (1.00-1.03)	.003	1.02 (1.00-1.04)	.048

*CI*, confidence interval; *OR*, odds ratio.

### Global analysis using a latent class model

To account for interrater variability and obtain a consensus estimate of the association between age and acromial morphology, a latent class model was subsequently applied.

This latent class analysis, integrating all ratings, demonstrated a significant association between age and the consensus classification of acromial morphology. The ORs for a 1-year increase in age were 1.02 for latent class 2 and 1.02 for latent class 3 compared with latent class 1 (Table V).

**Table V:** Latent class model: odds ratios per 1-year increase in age for acromions type 2 compared to type 1 (2/1) and type 3 compared to type 1 (3/1).

All raters		
Acromion type comparison	OR (95% CI)	*P* value
2/1	1.02 (1.01-1.04)	.008
3/1	1.02 (1.02-1.03)	.001

*CI*, confidence interval; *OR*, odds ratio.

**Figure 4:**
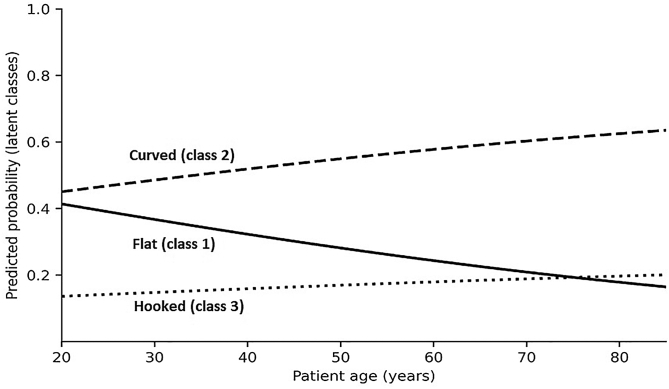
Distribution of the 3 latent acromial morphology classes according to age: flat (class 1), curved (class 2), and hooked (class 3).

The distribution of latent classes according to age is illustrated in Figure 4.

## Discussion

The Bigliani classification demonstrated good intrarater reliability but poor inter-rater reliability when applied to standardized 3D CT reconstructions. In addition, a significant association between acromial morphology and age was observed, suggesting that acromial shape may evolve over the course of life rather than representing a purely innate anatomical characteristic.

Previous studies evaluating the reliability of the Bigliani classification on standard radiographs, magnetic resonance imaging (MRI), and cadaveric specimens have already reported substantial inter-rater variability despite generally satisfactory intrarater reliability.^3^^,^^10^^,^^17^^,^^24^^,^^25^^,^^33^

Our findings are consistent with these observations and extend them to 3D CT imaging, which is frequently used in the pre-operative assessment of shoulder disorders.^18^^,^^19^

Despite optimized visualization of bony structures, the inter-rater reliability of this classification remains limited, highlighting its intrinsically subjective nature. Importantly, the use of standardized 3D CT reconstructions, which theoretically optimize visualization of osseous structures, did not improve inter-rater reliability. Nevertheless, the Bigliani classification continues to be widely used in clinical practice because of its simplicity and ease of application.

Given its widespread use, evaluating the reliability of this classification remains relevant because of its potential influence on clinical interpretation and decision-making.

Several alternative classifications and more objective morphological parameters have been proposed to improve the reliability of acromial assessment.^9^^,^^24^^,^^25^^,^^33^^,^^38^ Although some of these methods have demonstrated better inter-rater reliability, their clinical use remains limited, and their validation has mainly been restricted to standard radiographs.

The variability observed in previous studies using radiographs may be partly explained by the lack of standardized acquisition protocols, including variations in scapular positioning and projection angles. To date, no tool has emerged as a consensual and reproducible reference, particularly for CT imaging.

Regarding the relationship between acromial morphology and age, previous studies based on standard radiographs, MRI, cadaveric specimens, or histological analyses have reported conflicting results.^1^^,^^12^^,^^22^^,^^29^^,^^40^ Our findings, strengthened by a global analysis using a latent class model that accounts for inter-rater variability, provide additional evidence supporting age-related changes in acromial morphology. This approach enables estimation of a consensus classification by integrating variability between raters and reducing the impact of subjective disagreement. However, although statistically significant, the magnitude of this association remains modest.

These results should be interpreted cautiously, as the present study does not allow any inference regarding causal relationships between acromial morphology, aging, and shoulder pathology.

From a clinical perspective, the poor inter-rater reliability observed in this study suggests that the Bigliani classification should be interpreted with caution when used to inform treatment decisions.

Variability in its assessment may lead to divergent interpretations of impingement severity and, consequently, to different management strategies, particularly regarding acromioplasty, the indications for which remain controversial. Therefore, reliance on the Bigliani classification alone for clinical decision-making may be inappropriate, especially when guiding surgical indications such as acromioplasty.

These findings also have implications for research, as variability in classification may affect the comparability of studies using acromial morphology as a variable.

In this context, the present study should be interpreted as a methodological evaluation of a widely used classification system, focusing on its reliability based on osseous anatomy using standardized 3D CT reconstructions, rather than directly addressing its clinical application.

The association observed in this study between age and morphology reinforces the hypothesis of a partially acquired nature of acromial shape, challenging the concept of a fixed and innate morphology throughout life. These findings suggest that age-related morphological changes should be taken into account when interpreting imaging studies and their implications in the pathophysiology of subacromial disorders.

Future research should aim to develop more objective and reproducible tools for evaluating acromial morphology, potentially incorporating quantitative measurements or artificial intelligence–based approaches. Prospective multicenter studies combining morphological data with clinical outcomes could help clarify the role of acromial morphology in shoulder pathology and optimize therapeutic strategies, particularly by better defining the role of surgical treatment in a context where the effectiveness of acromioplasty remains debated.

### Strengths and limitations

The strengths of this study include the large sample size, the use of standardized high-resolution 3D reconstructions, blinded evaluations by multiple shoulder surgeons with varying levels of experience, and the use of a latent class statistical model to mitigate the impact of inter-rater variability in the analysis of the association between acromial morphology and age.

Several limitations should be acknowledged. The retrospective and single-center design may limit the generalizability of the findings. The Bigliani classification remains intrinsically subjective, contributing to the observed interrater variability.

In addition, the absence of clinical correlation prevents any interpretation regarding the relationship between acromial morphology, patient symptoms, or rotator cuff pathology.

Finally, the study design does not allow conclusions regarding causality between acromial morphology and age-related changes.

## Conclusion

Even with optimized CT imaging, the Bigliani classification demonstrated good intrarater reliability but poor interrater reliability, limiting its usefulness as a standardized tool for assessing acromial morphology.

A significant association between acromial morphology and age was identified, suggesting that acromial shape may evolve over time rather than represent a strictly innate anatomical characteristic.

These findings should be interpreted with caution, as this study was designed as a methodological evaluation of a widely used classification system rather than to establish clinical or causal relationships.

They also highlight the need for cautious interpretation of acromial morphology and for the development of more objective and reproducible assessment tools to better clarify its role in shoulder pathology.

## Disclaimers:

Funding: No funding was disclosed by the authors.

Conflicts of interest: The authors, their immediate families, and any research foundations with which they are affiliated have not received any financial payments or other benefits from any commercial entity related to the subject of this article.
